# Supplementary material for: The interactome of intact mitochondria by cross-linking mass spectrometry provides evidence for coexisting respiratory supercomplexes
Source: Mol Cell Proteomics. 2017 Dec 8;17(2):216–32. doi: 10.1074/mcp.RA117.000470 (PMC5795388; doi:10.1074/mcp.RA117.000470)
Supplement: Supplemental Data [file supp_RA117.000470_133922_0_supp_23978_fzffwf.zip › spectra_annotation/mito_DR_spectra_annotation/451-1-2-1-5-1.pdf]

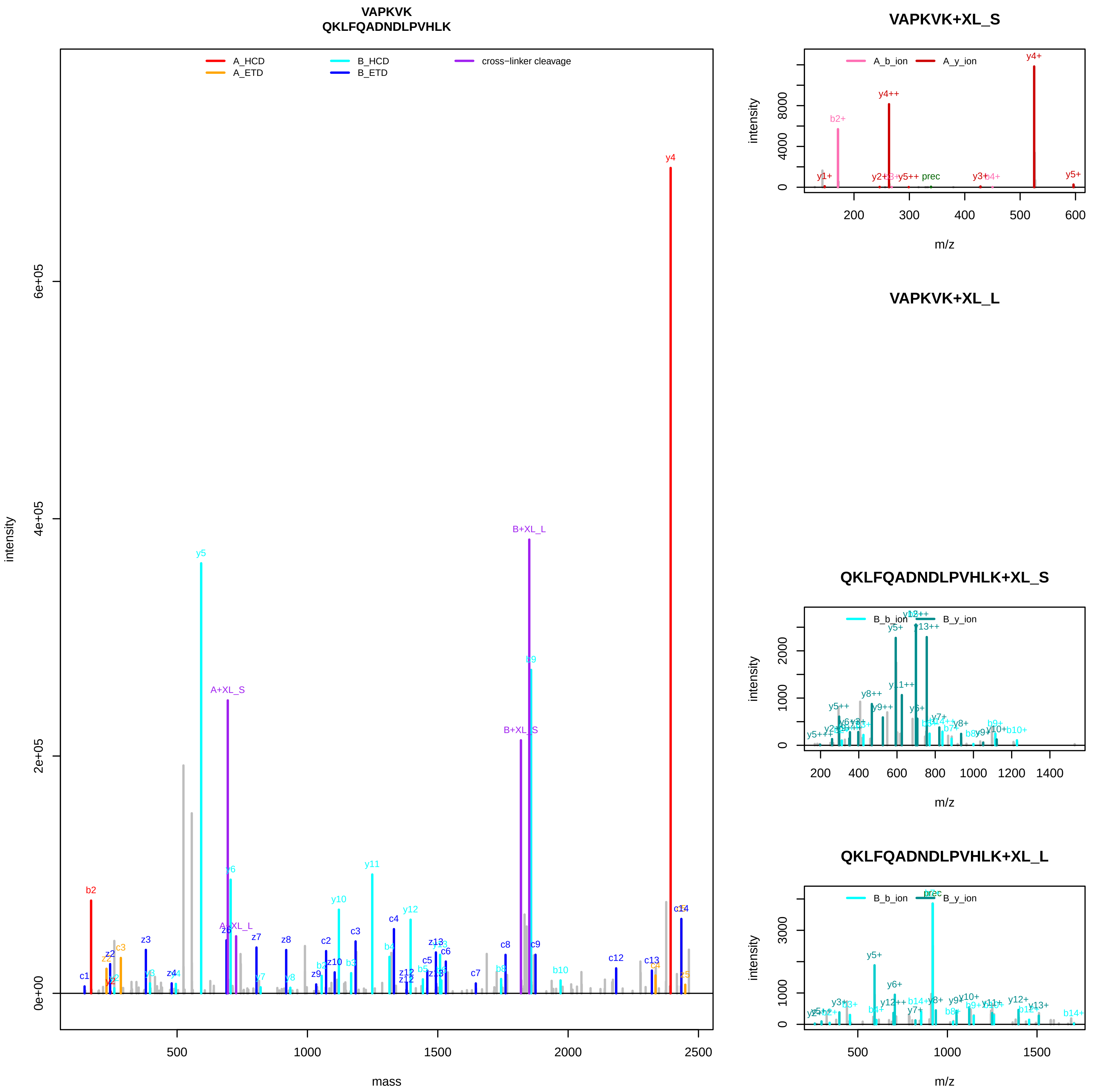
VAPKVK
QKLFQADNDLPVHLK
A_HCD
A_ETD
B_HCD
B_ETD
cross−linker cleavage
intensity
mass
0e+00
2e+05
4e+05
6e+05
500
1000
1500
2000
2500
y4
B+XL_L
y5
b9
A+XL_S
B+XL_S
y6
y11
b2
y10
y12
c14
A+XL_L
c4
z6
z7
z8
c2
c3
z13
y13
c6
c8
c9
c12
c13
b10
c7
VAPKVK+XL_S
A_b_ion
A_y_ion
y4+
y4++
b2+
y1+
y5++
prec
y3+
y5+
m/z
VAPKVK+XL_L
QKLFQADNDLPVHLK+XL_S
B_b_ion
B_y_ion
y12++
y5+
y13++
y8++
y11++
y9++
y5++
y7+
y8+
b9+
y10+
b10+
QKLFQADNDLPVHLK+XL_L
prec
y5+
y6+
y3+
y12++
b14+
y8+
y9+
y10+
y11+
y12+
y13+
b14+
